# Combination of Mechanical Treatment and Enzymatic Hydrolysis During Post‐Consumer Cotton Waste Processing

**DOI:** 10.1002/cssc.70698

**Published:** 2026-05-13

**Authors:** Miriam Magdalena Schaake, Oliver Pikhard, Moritz Bross, Tobias May, Zhi Cheng Hua, Luca Schmidt, Frank Kleine Jaeger, Andreas Liese, Stefan Heinrich

**Affiliations:** ^1^ Institute of Solids Process Engineering and Particle Technology Hamburg University of Technology Hamburg Germany; ^2^ Group Research BASF SE Ludwigshafen am Rhein Germany; ^3^ Institute of Technical Biocatalysis Hamburg University of Technology Hamburg Germany

**Keywords:** biocatalysis, dyed cotton waste, mechanoenzymatic processing, polymers, wet milling

## Abstract

With the continuously increasing volume of textile waste and the limitations of current recycling strategies, there is a growing need for the development of environmentally sustainable and efficient processing methods. Cellulose derived from post‐consumer textile waste represents a promising and cost‐effective substrate for enzymatic hydrolysis due to its abundance and low market value. This study investigates the synergistic effect of a cellulase enzyme mixture combined with wet rotor milling to enhance glucose yields during the enzymatic hydrolysis of cotton‐based textile waste. The impact of mechanical energy input is assessed by varying milling durations in the presence and absence of enzymes. Enzyme‐assisted milling enables a streamlined, single‐step process, increasing glucose yield by approximately 12% compared to conventional hydrolysis for 6 h. Two iterative cycles of milling followed by incubation in a feed tank are evaluated. The highest glucose conversion (38%) is achieved by combining a premilling step with iterative cycles of milling performed with minimal milling time and subsequent enzymatic hydrolysis. Extended milling times reduce enzymatic activity, suggesting potential inhibitory effects under certain conditions. Overall, the findings support that integrating enzymatic hydrolysis into milling operations is a viable strategy for the partial recycling and valorization of textile waste.

## Graphical abstract

This study introduces a combined mechanical and biological process for converting cotton textile waste into fermentable sugars. Textiles are preprocessed in a cutting mill and subsequently subjected to mechanoenzymatic treatment in a wet rotor mill. Iterative milling cycles enhance glucose yield, while extended milling reduces particle size without further improvement.

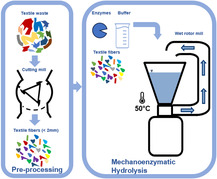

## 1 Introduction

The ongoing growth of the global population, combined with the continuous expansion of the fashion industry, has led to a substantial increase in textile waste generation [1, 2, 3]. This overproduction [4], along with the complex material composition of textiles, exerts considerable environmental pressure [5, 6], as the majority of discarded textiles are either disposed of in landfills or incinerated [7]. A considerable amount of this waste, estimated at approximately 24% [2, 8], is composed of cotton, which contains about 88–96 wt% cellulose [9, 10].

Given the scale of this waste stream, there is an urgent need for innovative technologies that enable mild and environmentally sustainable depolymerization of cellulose into glucose [11]. One promising approach for valorizing this cellulose rich material is enzymatic hydrolysis, which facilitates the conversion of cellulose into fermentable sugars. The resulting glucose can subsequently serve as a feedstock for various biotechnological applications, including the production of bioethanol and biogas [12, 13].

However, the structural complexity of cellulose, characterized by its high degree of polymerization and crystalline organization, presents a considerable barrier to enzymatic accessibility [14, 15]. Nevertheless, the implementation of specific pretreatment methods can facilitate the depolymerization of cellulose and reduce its crystallinity. However, these methods often entail high energy consumption or rely on harsh chemical conditions, which pose environmental and economic concerns [11, 16].

Recent studies have demonstrated that enzymes can retain their activity even under substantial mechanical stress [17, 18]. Specifically, efficient mechanoenzymatic hydrolysis of cellulose has been achieved through iterative cycles of milling followed by static incubation of physical mixtures composed of solid substrates, freeze‐dried cellulase preparations, and minimal water content. Upon initiation of enzymatic hydrolysis, the process alternates between brief periods of mechanical energy input and extended resting phases. This innovative process, termed reactive aging (RAging), has resulted in significantly higher glucose concentrations compared to previously reported methods, notably without the necessity for pretreatment or the application of aggressive chemicals [17, 18].

Another phenomenon that facilitates the processing of biological systems is known as liquefaction [19]. This phenomenon leads to a reduction in the viscosity of the suspension [20], thereby improving its pumpability and enabling higher solid loadings, which are critical parameters for the efficient operation of bioconversion processes at industrial scale [20, 21]. In this regard, the addition of enzymes has been shown to promote system fluidization, which in turn enhances the overall processability of biomass substrates. The influence of total solids content on the rheological behavior of cellulose and lignocellulosic suspensions has been extensively studied, particularly in the context of optimizing enzymatic hydrolysis [22].

Recent work identified wet rotor milling as a promising pretreatment method to enhance the enzymatic accessibility of end‐of‐life cotton textiles [23]. Building upon these findings, the present study examines the combined application of wet rotor milling and a cellulase enzyme mixture, with hydrolytic reactions occurring simultaneously during mechanical processing, to improve glucose release from cotton‐based textile substrates. The investigation focuses on the effect of varying milling durations on enzymatic conversion efficiency under technical‐scale conditions. To ensure experimental consistency and comparability, white cotton textiles with a cellulose content representative of the target waste material were employed. The resulting glucose yields served as a quantitative benchmark for assessing the performance of the mechanoenzymatic treatment. For clarity and consistency, the term dyed cotton waste will be used throughout this study to refer to end of life cotton textiles.

## 2 Results and Discussion

The following sections present evidence that mechanoenzymatic processing of textile materials enhances saccharification efficiency. The effectiveness of the applied process strategies was assessed using a combination of analytical techniques, including near‐infrared (NIR) spectroscopy, crystallinity measurements, particle size distribution analysis, viscosity determination, and quantification of glucose yield resulting from enzymatic hydrolysis. More detailed information on the operation of the wet rotor mill is available in the Supporting Information and in the preceding publication [23]. Four processing options are presented in Table 1. The RAging cycles employed included 1 min of milling followed by 29 min of resting, as well as a cycle of 0.16 min of milling followed by 29.84 min of resting.

**TABLE 1:** The experimental process options include a range of comminution processes conducted at solid loadings of 5 wt% and a gap width of 350 μm, during which enzymatic degradation took place using CTec2 at a temperature of 50°C and a pH of 4.8, with an enzyme concentration of 25 FPU/g maintained over a period of 6 h. In process options 3 and 4, a milling step was included prior to enzyme addition.

Process	Material	Energy input before enzymatic hydrolysis, kWh/t	RAging cycle
1: No Milling (NoMi)	Dyed cotton waste White cotton	—	—
2: RAging (RA)	Dyed cotton waste White cotton	—	1/29 min
3: Milling + RAging (MiRA 1)	Dyed cotton waste	85	0.16/29.84 min
	White cotton		
4: Milling + RAging (MiRA 2)	Dyed cotton waste	85	1/29 min

### 2.1 Determination of Digestible Cellulose Fraction

NIR spectroscopy measurements indicated a cellulose content of 94 wt% in both dyed cotton waste and white cotton, with standard deviations of 7.93 and 8.79, respectively. These results suggest that approximately6 wt% of the material may be resistant to enzymatic hydrolysis under applied conditions. To ensure accurate mass balance calculations during enzymatic hydrolysis and to enable a consistent assessment of process efficiency, a cellulose content of 94 wt% was used as the basis for calculating the glucose yields presented in the following sections.

### 2.2 Influence of Milling and Enzymatic Hydrolysis on Particle Size and Physical Properties

**FIGURE 1:**
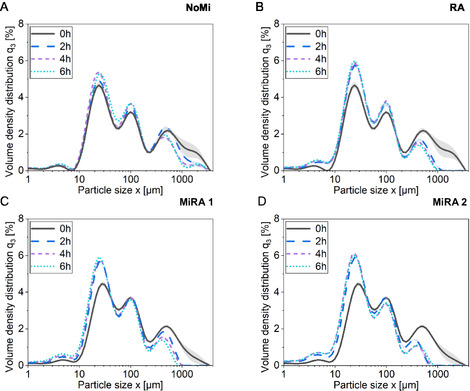
The effect of mechanoenzymatic processing on particle size distribution was investigated. Samples were collected at 0, 2, 4, and 6 h during the 6 h trial period. Four distinct processing conditions were evaluated for dyed cotton waste: (A) No milling (NoMi), (B) RAging cycle 2 (RA), (C) milling + RAging cycle 1 (MiRA 1), and (D) milling + RAging cycle 2 (MiRA 2).

The influence of mechanical energy in combination with enzymatic hydrolysis on particle size and physical properties was investigated, with the results presented in Figure 1 and Table 2. Four distinct processing strategies using dyed textile waste were evaluated based on their particle size distributions. Initial samples were collected prior to enzymatic treatment, meaning that in two of the process variants, an additional milling step had already been applied. The parameter *x*_90,3_ refers to the particle size below which 90% of the total particle volume is contained, while *x*_50,3_ denotes the volume based median diameter.

**TABLE 2:** The effect of mechanoenzymatic processing on particle size was evaluated by reporting *x*_50,3_ [µm] and *x*_90,3_ [µm] values at the initial time point (0 h) and after 6 h (6 h) of treatment. Four distinct processing conditions were evaluated for dyed cotton waste: (A) No milling (NoMi), (B) RAging cycle 2 (RA), (C) milling + RAging cycle 1 (MiRA 1), and (D) milling + RAging cycle 2 (MiRA 2).

	A: NoMi		B:RA		C: MiRA 1		D: MiRA 2	
—	*x*_50,3_, µm	*x*_90,3_, µm	*x*_50,3_, µm	*x*_90,3_, µm	*x*_50,3_, µm	*x*_90,3_, µm	*x*_50,3_, µm	*x*_90,3_, µm
*t*_0_	74.80	757.21	74.80	757.21	77.33	634.61	77.33	634.61
*t*_6_	53.77	489.61	37.66	249.85	37.44	219.05	33.24	165.81

Table 2 illustrates that 6 h enzymatic hydrolysis without mechanical treatment decreases the *x*_50,3_ value from 74.80 to 53.77 μm, indicating a moderate yet measurable size reduction. In contrast, MiRA 1 and RAging exhibit comparable *x*_50,3_ and *x*_90,3_ values, with only slight changes observed across pre‐ and post‐treatment conditions. However, the MiRA 2 treatment, characterized by an extended milling period and higher energy input, induces a noticeable shift in particle size distribution (Figure 1D), reducing the *x*_90,3_ value from 634.61 to 165.81 μm (Table 2). This contrasts with MiRA 1 (Figure 1C), which applies to a shorter RAging cycle and reduced milling intensity. The higher energy input is assumed to contribute to the more pronounced reduction. Through enzymatic hydrolysis, a marked reduction in the coarse fraction is observed (Figure 1). This decrease may be caused by disintegration of agglomerates due to enzymatic hydrolysis. Supporting this hypothesis, a decrease in the coarse fraction is also detected in the absence of mechanical energy input. However, the introduction of mechanical energy significantly improves this effect, particularly during the first 2 h of enzymatic hydrolysis. It clearly shows that there is a positive dependence between energy input and smaller particle sizes, which is supported by the inducing of enzymes. Furthermore, the reduction in fiber size due to fiber breakage and enzymatic degradation is clearly pronounced across all process options involving mechanical energy input. Concurrently, an increase in the fine fraction becomes apparent, which could suggest a higher proportion of partially comminuted and enzymatically degraded fibers. Studies indicate that greater refining intensity, corresponding to increased energy input, during biomass processing results in continuous particle size reduction [24]. Furthermore, mechanical refining of biomass has been demonstrated to correlate with improved enzymatic yield as particle size decreases [25, 26]. A similar effect has been observed when biomass is pretreated using a hammer mill [27]. Additionally, the literature describes a reduction in particle size during enzymatic hydrolysis [28].

Achieving precise fiber size measurements continues to be a methodological challenge. Microscopic imaging is commonly utilized as technique [29, 30], providing a compromise between measurement duration, image resolution, and magnification flexibility for size characterization, including potential industrial applications [30]. A major limitation arises from the nature of textile fibers, which makes imaging‐based approaches resource intensive [29, 31]. Fiber analyzers commonly employed in the paper industry operate within a size range of 0.2–5 mm. For more detailed investigations, a change in instrumentation would be necessary to accommodate different size ranges [30, 32]. Consequently, laser diffraction was selected in this study, as it is widely utilized across various fields and offers distinct advantages, including rapid measurement and broad applicability. However, it should be emphasized that, due to inherent measurement variability [33], the outcomes are best interpreted on a relative basis rather than as absolute values. Nevertheless, the data reveal a consistent trend that is supported by complementary measurements.

Visual inspection of the scanning electron microscopy (SEM) images (Figure S1) reveals no significant differences in surface morphology among the treated fiber samples.

With the exception of the untreated crude textile, all acquired images exhibit a relatively uniform and smooth fiber surface. During wet milling, intact fibers undergo fragmentation, which may subsequently lead to structural modifications such as delamination or fibrillation [34]. These observations suggest that, despite the different processing conditions applied, the surface morphology of the fibers remains largely consistent across treatments.

The effects of various process strategies on cellulose crystallinity are summarized in Table 3. Information on the adsorption ratio calculation is provided in the Methods section. Our findings indicate a distinct and consistent trend: enzymatic hydrolysis leads to an increase in crystallinity, whether applied independently or in combination with mechanical pretreatment. The extent of crystallinity enhancement, as presented in the subsequent results, is strongly influenced by the specific process configuration.

**TABLE 3:** Crystallinity index obtained from FTIR absorption ratios. Samples from the No milling, milling + RAging 1 (MiRA 1), milling + RAging 2 (MiRA 2), and RAging (RA) conditions were collected following 6 h of enzymatic hydrolysis.

Sample	LOI (*A*_1430_/*A*_897_)	HBI (*A*_3336_/*A*_1336_)	CCI (*A*_1278_/*A*_1263_)
No Milling	1.07 ± 0.03	1.51 ± 0.08	1.13 ± 0.01
RAging	1.11 ± 0.04	1.53 ± 0.05	1.15 ± 0.01
MiRA 1	1.14 ± 0.01	1.62 ± 0.10	1.16 ± 0.02
MiRA 2	1.17 ± 0.07	1.62 ± 0.02	1.16 ± 0.01

This study builds on previous research that focuses solely on crystallinity after mechanical processing. Under conditions of mechanical treatment with a gap width of 350 µm and a solids loading of 5%, crystallinity varies depending on the applied energy input (55–200 kWh/t), ranging from 0.63 to 0.70 (LOI), 1.22–1.27 (HBI), and 1.10–1.12 (CCI) [23]. Compared with these results, enzymatic hydrolysis without prior milling leads to an increase in crystallinity; however, this increase is less pronounced compared to the process variants involving MiRA 1 and MiRA 2. These latter approaches yield identical crystallinity values for HBI (1.62) and CCI (1.16), suggesting that the additional longer milling time does not result in further significant changes in crystallinity. In contrast, the LOI method indicates a slight increase in crystallinity from 1.14 (MiRA 1) to 1.17 (MiRA 2). This observation aligns with the findings presented in subsequent sections. Since a reduction in crystallinity facilitates enzymatic degradation, this leads to an increased glucose yield [35]. Therefore, it can be concluded that among the various process configurations investigated, the combination of mechanical and enzymatic pretreatment, particularly the MiRA 2 process, has the most pronounced effect on crystallinity.

It is important to note that the literature reports varying outcomes regarding changes in crystallinity following enzymatic hydrolysis. While substrates such as Avicel [14] and switchgrass [36] show no significant change in crystallinity, studies involving cotton and post‐consumer textiles report an increase in crystallinity after enzymatic degradation [35, 37]. The literature demonstrates that cellulases are capable of quantitatively depolymerizing cellulose, including the degradation of crystalline regions. This process becomes particularly effective when cellulases act synergistically with additional enzymes [38]. Current mechanistic interpretations propose a sequential, layer‐wise degradation process whereby external cellulose layers are hydrolyzed first, gradually exposing inner domains and facilitating the breakdown of both amorphous and crystalline fractions [39]. Nevertheless, an increase in crystallinity is commonly observed in the initial hydrolysis phase, attributed to the preferential and faster removal of amorphous regions relative to crystalline ones [38, 40]. The correlation between crystallinity and glucose yield has been confirmed in other studies [14, 23].

### 2.3 Influence of Enzymatic Hydrolysis on Rheological Properties

**FIGURE 2:**
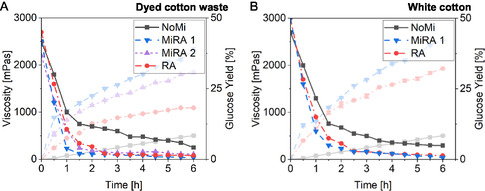
The effect of mechanoenzymatic processing on rheological properties of dyed cotton waste (A) and white cotton (B) was investigated using four distinct treatment protocols: milling combined with RAging cycle 1 (MiRA 1), RAging cycle 2 (RA), milling combined with RAging cycle 2 (MiRA 2), and a control without milling (NoMi). Each protocol was applied over a 6 h period, during which samples were collected every 30 min for subsequent analysis. The glucose curves for the respective process variations are presented in a lighter tone.

The rheological properties of textile suspensions in combination with enzymatic hydrolysis were investigated to characterize the material influence on glucose production. Figure 2 illustrates the viscosity reduction observed in textile fiber suspensions in different process options.

The enzymatic hydrolysis process results in a consistent decrease in viscosity across all suspensions. Notably, a similar pattern of viscosity reduction is evident regardless of the pretreatment applied. The mechanical energy imparted during the subsequent hours of the experiment has a significantly positive effect on viscosity reduction, as demonstrated by the observation that the sample lacking pretreatment exhibits the least reduction in viscosity. It is likely that the minimum viscosity is achieved when substrate fibers are hydrolyzed to their smallest sizes. The enzymatic action predominantly targets the amorphous fibers present on the surfaces of the particles, leading to a decrease in entanglement among adjacent particles [22]. Particle fragmentation, reduction of interparticle surface interactions, and material dilution are recognized as the primary mechanisms facilitating viscosity reduction. Importantly, viscosity reduction occurs prior to cell hydrolysis and correlates with the fragmentation observed, suggesting the existence of a distinct liquefaction step preceding the saccharification process [20].

Similar rheological behavior has been documented in studies involving corn stover pellets [21]. The reductions in viscosity are believed to reflect changes in the structure of long‐chain molecules within fibrous particles [19]. Additionally, literature indicated that viscosity reduction in lignocellulosic structures is associated with degradation and a decreased capacity for water binding during enzymatic activity [22]. Throughout the conversion process, the rheological properties of the slurry undergo significant and dynamic changes as various chemical bonds within the solid phase of the biomass are hydrolyzed, leading to the solubilization of components into the liquid phase.

### 2.4 Influence of Mechanoenzymatic Processing

**FIGURE 3:**
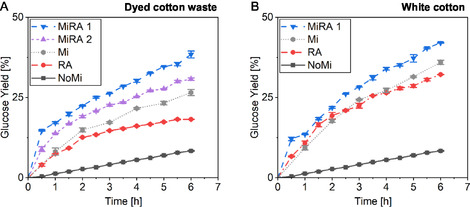
The effect of mechanoenzymatic processing on the conversion of dyed cotton waste and white cotton into glucose was investigated using four distinct treatment protocols: milling combined with RAging cycle 1 (MiRA 1), RAging cycle 2 (RA), milling combined with RAging cycle 2 (MiRA 2), and a control without milling (NoMi). Each protocol was applied over a 6 h period, during which samples were collected every 30 min for subsequent analysis. An additional curve (Mi) was included to represent a sample that underwent milling without enzymes, followed by enzymatic hydrolysis conducted at a different scale (see Supporting Information Section 2.3.2). (A) Dyed cotton waste (B) White cotton.

The influence of mechanical energy of textile suspensions in combination with enzymatic hydrolysis was investigated. Results can be seen in Figure 3. Four distinct process configurations involving dyed textile waste and white cotton were evaluated over a 6 h trial period. To enable comparative analysis, the effects of RAging (RA) and the absence of milling were also investigated. Previous experimental results indicated a clear trend in favor of white cotton with respect to performance outcomes [23]. Based on these results, the MiRA 2 trial involving white cotton was discontinued to enable a more efficient distribution of resources toward alternative variants. Control experiments further demonstrated that mechanical processing under the applied conditions alone did not affect glucose formation. In the absence of enzymes, neither the unmilled reference nor the wet‐milled sample (dyed cotton waste, 350 µm, 5 wt%, 85 kWh/t) showed any detectable glucose release in triplicate analyses.

Enzymatic degradation without any additional mechanical treatment yielded a glucose concentration of 8%. In contrast, a combined approach involving 1 min of milling followed by 29 min of resting (RA) significantly enhanced the glucose yield from dyed textiles, reaching 18%. When RA was applied to white cotton, it resulted in a significantly higher glucose yield (32%) compared to dyed textile waste. This discrepancy is likely attributable to the inhibitory influence of dye compounds on enzymatic activity, as previously reported [41]. Nonetheless, the application of RA markedly improved overall yields, potentially due to the additional energy input introduced during the process.

The milling parameters and enzyme activity (25 FPU/g) were established in a previous study [23], which identified an energy input of 85 kWh/t, a rotor‐stator gap width of 350 µm, and a solids loading of 5% as optimal conditions for enzymatic hydrolysis during wet milling. Under these parameters, extending the milling duration in the absence of cellulases consistently enhanced glucose yields from both white and dyed cotton substrates. Based on these findings, the process variant involving prior milling followed a short cycle of RAging (MiRA 1) demonstrated the highest overall performance. It is important to emphasize that the reported duration represents the minimum residence time applied in this experiment. The material underwent minimal pass through the system. A trial involving premilling followed by enzymatic degradation could not be conducted due to the inability to ensure adequate mixing of enzymes within the feed pipe. Instead, pretreatment was conducted using a wet rotor mill (350 µm, 5%, 85 kWh/t), followed by enzymatic hydrolysis (5 wt%, 25 FPU/g) at a different scale. The trials resulted in glucose yields of 27% for dyed cotton waste and 36% for white cotton after 6 h. In comparison, MiRA 1 appears to deliver significantly improved outcomes. After 6 h of enzymatic treatment, this approach achieved a glucose yield of 38% for dyed cotton waste and 42% for white cotton. Comparison with similar pretreatment methods (Mi) demonstrated that the selected process configuration markedly enhances glucose yield. However, since this type of mill has only been employed for textile pretreatment in own publication [23], a direct comparison with existing literature is limited. The following comparison relies on data from other milling configurations and materials. Hammer milling yielded up to 19% glucose after 6 h of enzymatic hydrolysis of cotton textiles [42], while ball milling of lignocellulosic biomass achieved approximately 30% under the same conditions [43]. However, comparison with alternative treatment strategies applied to dyed cotton waste reveals that additional improvements could be achievable. By contrast, pretreatment of textiles using a sodium hydroxide/urea mixture produced glucose yields below 25% after 8 h of enzymatic hydrolysis [44], whereas acid hydrolysis achieved yields of up to 50% after 6 h [45].

**FIGURE 4:**
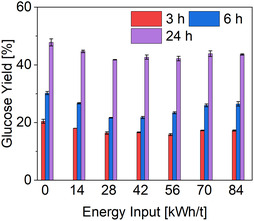
To isolate the effect of mechanical energy on enzyme stability, the buffer enzyme suspension was processed without any textiles using a cyclic milling protocol (RA cycle 2) over a 6 h period, with samples collected every 30 min. Following this step, the mechanically stressed enzyme samples were evaluated in a separate enzymatic hydrolysis experiment using milled dyed cotton waste (85 kWh/t). Hydrolysis was conducted with CTec2 at 50°C and pH 4.8, at a solids load of 2.5 wt% and an enzyme dosage of 50 FPU/g textile over a 24 h period. Samples were collected at 0, 3, 6, and 24 h.

Although milling positively influenced enzymatic degradation, the combined application of milling and RAging 2 (MiRA 2) resulted in reduced yields (31%) for dyed cotton waste. These observations indicate that longer milling time may have an inhibitory effect on enzymatic activity under certain conditions. Previous studies have demonstrated that cellulase mixtures can be effectively applied during milling processes. Mechanoenzymatic approaches, which combine mechanical and enzymatic treatments, have proven successful for a variety of enzymatic reactions [46]. These methods typically operate under high solid loadings and utilize different types of milling equipment, including ball mills, roller mills, and planetary ball mills [46, 47]. For comparative purposes, studies have shown that a cycle consisting of 5 min of milling followed by 55 min of resting can significantly enhance enzymatic degradation [48]. To further investigate the potential inhibitory effects of wet rotor milling on enzyme activity, additional trials were conducted using a buffered enzyme suspension under identical process conditions. Samples were taken at 30 min intervals over a 6 h period and subsequently subjected to enzymatic hydrolysis for 24 h. The corresponding results are presented in Figure 4. Control measurements were performed at the beginning of the study to account for baseline signals from the enzyme solution. Because Cellic CTec2 contains a measurable amount of glucose, its intrinsic glucose content was quantified using textile free reference samples and subtracted from the total measurements. The 0 h sample is not shown because its value was zero after correction and was incorporated into the baseline adjustment.

The absence of milling resulted in the highest glucose yield, reaching 48% after 24 h of enzymatic hydrolysis. Mechanical agitation and hydrodynamic shear are known to induce partial or complete deactivation of proteins [49, 50], including cellulolytic enzymes [51, 52]. This is consistent with the experimental findings showing reduced hydrolysis efficiency after wet‐rotor milling compared with unmilled controls. Across all cellulase preparations tested, milling exerted an inhibitory effect on enzymatic activity, although the extent of inhibition remained relatively moderate. Even minimal energy input appeared sufficient to reduce the activity of the cellulase mixture, although the variability in the results was considerable. The resulting glucose yields ranged from approximately 45% (at 14 kWh/t) to 44% (at 84 kWh/t). Nonetheless, it is important to emphasize that the inhibitory effect does not correlate with the level of mechanical energy applied. To compare these results with those presented in Figure 3, it is important to consider that additional factors may influence the observed glucose yields. One possible explanation is the liquefaction of the textile suspension, as shown in Figure 2. As the viscosity of the suspension decreases, the efficiency of the milling process is progressively reduced. Although milling typically facilitates fiber accessibility by applying mechanical forces, a lower viscosity can limit the interaction between the textile fibers and the rotor. As the medium becomes more fluid, the mechanical stress exerted on the fiber's decreases, thereby reducing the intensity and effectiveness of shear forces required for efficient fiber disruption. Such an explanation may also clarify why intensified energy input during wet milling in buffered enzyme systems leaves enzymatic activity largely unaffected. Milling in a wet rotor mill is most effective during the initial passes [53]. Beyond this point, no further increase in surface area is achieved, leading to a plateau in enzymatic degradation efficiency. At lower solid concentrations, particle morphology becomes increasingly important. It can be concluded that particle size is a key parameter affecting slurry rheology, particle disintegration, and the efficiency of liquefaction under low solid content conditions [20]. Since solids loading is a critical factor in the liquefaction process, it is essential to identify an optimal balance between milling parameters, solids concentration, and the timing of enzyme addition. Enzymatic hydrolysis of cotton textiles at high solids concentrations could lead to improved glucose yields and, consequently, increased ethanol production during fermentation. Enzyme‐mediated liquefaction represents a promising strategy for converting solid biomass into pumpable slurries [21, 22]. In this context, the implementation of high solids saccharification is also essential to further improve the overall efficiency and cost‐effectiveness of cellulose conversion.

The sustainability of the proposed mechaoenzymatic treatment requires critical evaluation, as mechanical approaches are consistently described in the literature as economically challenging due to their high energy demand [41, 54, 55]. Acidic and alkaline pretreatments likewise involve considerable operational costs for chemicals, recovery technologies, and extended residence periods [41]. In contrast, the minimal mechanical energy input applied here reveals that a short, targeted energy impulse during hydrolysis can enhance processability while remaining compatible with scalable equipment configurations.

Despite the enhanced glucose yields, the extent to which these improvements offset the additional energy input remains uncertain. Further optimization strategies, such as fed‐batch operation with substrate addition or extended reaction times, may offer additional potential to increase glucose yields. However, a robust techno‐economic and lifecycle assessment will be essential to determine whether energetic investment is justified.

## 3 Conclusion

This study explored the synergistic effects of mechanical and enzymatic processing on dyed cotton waste. Integrating enzymatic hydrolysis during milling (MiRA 1) yielded the highest glucose conversion (38%), while extended milling prior to hydrolysis (MiRA 2) reduced yield (31%), suggesting potential enzyme inhibition. Rheological analysis showed a consistent viscosity decrease across all treatments, independent of pretreatment. Particle size distribution and SEM imaging confirmed fiber fragmentation and morphological changes due to enzymatic degradation. These findings highlight the interplay between fiber structure, rheology, and saccharification efficiency. Integrating enzymatic hydrolysis into milling operations is a promising strategy for textile waste valorization. However, reduced enzymatic activity under mechanical stress underscores the need for process optimization. A fed‐batch approach with incremental substrate addition is proposed to enhance liquefaction, reduce energy input, and improve economic viability. Achieving high substrate loading while maintaining enzymatic efficiency is key to maximizing overall process performance. Upcoming studies will examine extended reaction periods to enable more complete cellulose hydrolysis to glucose. The glucose obtained through this process presents opportunities for use in downstream fermentation applications.

## 4 Experimental Section

Detailed information on all materials and experimental procedures is provided in the Supporting Information (Appendix: Experimental Details)

### 4.1 Materials

The dyed cotton waste samples were obtained from ModaRe (Caritas, Madrid, Spain). For comparative analysis, a second substrate, commercially available white cotton (ATM Handel & Service GmbH, Winsen/Luhe, Germany) was used. Prior to mechanical pretreatment, the material was manually cut into pieces of uniform size. The cellulose content of all textile samples was verified using NIR spectroscopy.

### 4.2 Methods

#### 4.2.1 Mechanical Pretreatment

##### 4.2.1.1 Dry Shredding

Precutting was conducted using a Pallmann PS 3 ½ cutting mill (Pallmann, Zweibrücken, Germany). Subsequently, a Wanner C17.26s cutting mill (Wanner Technik, Wertheim, Germany). The target cut size was achieved using a 2 mm conidur sieve (Hein, Lehmann GmbH, Krefeld, Germany).

##### 4.2.1.2 Wet Milling

A Fryma MZ 80 wet rotor mill (FrymaKoruma, Rheinfelden, Germany) regulated via the IKA Eurostar 60 control system (IKA, Staufen im Breisgau, Germany) was employed. The gap width was adjusted to 350 μm, and a solids load of 5 wt% was tested. Both the feed tank and the feed pipe were connected to a MAGIO MS‐bc 4 circulation thermostat (Julabo, Seelbach (Schütter), Baden‐Württemberg, Germany) to maintain temperature control of the suspension.

#### 4.2.2 Analysis of Product Properties

##### 4.2.2.1 Crystallinity Measurement

To assess the crystallinity of cotton samples, Fourier‐transform infrared (FTIR) spectroscopy measurements were used. The measurements were executed in triplicate, and the results were averaged. The analysis involved calculating the ratios between the absorption peaks at 1430 and 897 cm^−1^, known as the lateral order index (LOI), as well as the ratios at 3336 and 1336 cm^−1^ (hydrogen bond intensity, HBI), and 1278 and 1263 cm^−1^ (Carrillo‐Colom index, CCI). These ratios were utilized to calculate the infrared crystallinity index (CRI). Further information can be found in previous publication [23].

##### 4.2.2.2 Particle Size Distribution Analysis

The particle size distribution of the textile suspension was assessed in at least triplicate using the wet dispersion unit of the Mastersizer 3000 (Malvern Panalytical, United Kingdom) for laser diffraction measurements. To effectively mitigate settling or separation of the sample in the aqueous dispersant, a stirrer operating at a speed of 2000 rpm was employed. The concentration of the sample was regulated through the obscuration parameter, which was maintained within the range of 5%–20%. Particle size distribution was determined using the Mastersizer 3000 software on Fraunhofer scattering theory.

##### 4.2.2.3 Yield Stress

Viscosity of textile suspensions was determined during enzymatic hydrolysis every 30 min for 6 h using a HAAKE Viscotester 3 rotational viscometer (Thermo Fisher Scientific, Karlsruhe, Germany) measuring the shear stress during deformation at a shear rate of 62.5 min^−1^. Measurements were made in measurement cup 3 (90 mm diameter, 75.5 mm height) using rotor no. 3 (45.1 mm diameter, 47 mm height) at room temperature (21°C).

#### 4.2.3 FTIR (Fourier Transform Infrared Spectroscopy)‐Analysis

FTIR spectroscopy was conducted using a Vertex 70 spectrometer (Bruker Optik GmbH, Ettlingen, Germany), which was equipped with a single‐bounce diamond attenuated total reflectance (ATR) insert (MIRacle, Pike Technologies) and a liquid‐cooled MCT detector. The Vertex 70 spectrometer was continuously flushed with dry, carbon dioxide‐free air. Spectra were acquired with 64 scans per spectrum, covering the range from 4000 to 600 cm^−1^, utilizing the OPUS 7.0 software package (Bruker Optik GmbH, Ettlingen, Germany). All samples were tested under dry conditions. Each spectrum underwent preprocessing, which included baseline correction and area normalization. To reduce the impact of temporal baseline fluctuations, a background spectrum was recorded against air prior to conducting the measurements.

#### 4.2.4 SEM

SEM images of the textile fiber were recorded utilizing a Zeiss Supra 55 VP to characterize morphology. All images were taken at an accelerating voltage of 3 kV. Samples were collected following each trial and subsequently dried for a duration of 3 days in a drying oven (Heraeus, Hanau, Germany) at a temperature of 50°C. A qualitative analysis was conducted to investigate the microstructural changes resulting from the mechanoenzymatic pretreatment.

#### 4.2.5 Enzymatic Hydrolysis

A commercial cellulase formulation, Cellic CTec2 (Novonesis, provided by Sigma–Aldrich, SAE 0020–50 mL) was employed for the enzymatic hydrolysis of cotton textiles. The hydrolysis was carried out in a 50 mM citrate buffer at pH 4.8, with 1 g/L of polyethylene glycol (PEG 6000, AnalytiChem, Eschborn, Germany) at a temperature of 50°C. An enzyme concentration of 25 FPU per gram of substrate was applied. Enzyme activity was determined using the FPU assay [56], and glucose concentration was measured with a Cedex Bio Analyzer 3D Lab (Roche, Basel, Switzerland).

## Supporting Information

Additional supporting information can be found online in the Supporting Information section. The authors have cited additional references within the Supporting Information ^[^ ^13^ ^,^ ^23^ ^,^ ^56^, ^57^, ^58^, ^59^, ^60^ ^]^.

## Funding

This study was supported by BASF.

## Conflicts of Interest

The authors declare no conflicts of interest.

## Supporting information

Supplementary Material

## Data Availability

The data that support the findings of this study are available from the corresponding author upon reasonable request.
