# Supplementary material for: Combination of Mechanical Treatment and Enzymatic Hydrolysis During Post‐Consumer Cotton Waste Processing
Source: ChemSusChem. 2026 May 13;19(9):e70698. doi: 10.1002/cssc.70698 (PMC13170930; doi:10.1002/cssc.70698)
Supplement: Supplementary file 1 — Supplementary Material [file CSSC-19-e70698-s001.pdf]

Supporting Information

Combination of Mechanical Treatment and Enzymatic Hydrolysis during Post-Consumer Cotton Waste Processing

Miriam Magdalena Schaake*[a,b], Oliver Pikhard[b], Moritz Bross[b], Tobias May[b], Zhi Cheng Hua[a], Luca Schmidt[c], Frank Kleine Jaeger[b], Andreas Liese[c], Stefan Heinrich[a]

[a] M.M. Schaake, Z.C. Hua, Prof. S. Heinrich
Institute of Solids Process Engineering and Particle Technology
Hamburg University of Technology
Denickestraße 15, 21073, Hamburg, Germany
E-mail: Miriam.Schaake@tuhh.de

[b] M.M. Schaake, Dr. O. Pikhard, Dr. M. Bross, Dr. T. May, Prof. F. Kleine Jaeger,
Group Research
BASF SE
Carl-Bosch-Straße 38, 67056, Ludwigshafen am Rhein, Germany

[c] L.Schmidt, Prof. A. Liese
Institute of Technical Biocatalysis
Hamburg University of Technology
Denickestraße 15, 21073, Hamburg, Germany

Table of Contents

Experimental Details 2
1 Materials 2
2 Methods 2
2.1 Analysis of Cellulose Content 2
2.2 Mechanical Pretreatment 2
2.2.1 Dry Shredding 2
2.2.2 Wet Milling 2
2.3 Analysis of Product Properties 3
2.3.1 Determination of Enzyme Activity 3
2.3.2 Enzymatic Hydrolysis 3
2.3.3 Glucose Measurement 4
Figure S 1 5
References 6

Experimental Details

1 Materials

The cotton textile waste samples were obtained from ModaRe (Caritas, Madrid, Spain) and manually presorted to isolate a cotton-rich fraction representative of materials that are available at industrially relevant scales and economically feasible prices. For comparative analysis, a second substrate, commercially available white cotton (ATM Handel & Service GmbH, Winsen/Luhe, Germany) was used. Prior to mechanical pretreatment, the material was manually cut into pieces of uniform size. The cellulose content of all textile samples was verified using near infrared (NIR) spectroscopy.

2 Methods

2.1 Analysis of Cellulose Content

Near-infrared spectroscopy (NIR) was conducted utilizing a SYS-IR-R-P spectrometer (TrinamiX GmbH, Ludwigshafen, Germany), operating within a spectral range of 1430 - 2500 nm and a wavenumber range of 4000 to 7000 cm^{-1}. The detector employed was a 256-pixel PbS line-array detector. A total of 50 samples were analyzed to determine the cotton content, and the results were averaged. This analysis was conducted for both used textiles and white cotton, with each sample having a mass of 1 kg.

2.2 Mechanical Pretreatment

2.2.1 Dry Shredding

Pre-cutting was conducted using a Pallmann PS 3 ½ cutting mill (Pallmann, Zweibrücken, Germany). The machine operated at a rotational speed of 715 rpm, yielding a circumferential speed of 12 m/s, with a rotor diameter of 320 mm. A two-step methodology was employed, utilizing two perforated plate sieves with a mesh size (square openings) of 15 mm and 8 mm, respectively. The feed rate during this process was set at 20 kg/h.

Subsequently, a Wanner C17.26s cutting mill (Wanner Technik, Wertheim, Germany) with a 3 kW motor was utilized to further reduce the material to pieces smaller than 2 mm. This target cut size was achieved using a 2 mm conidur sieve (Hein, Lehmann GmbH, Krefeld, Germany). The rotation speed of this mill was maintained at 380 rpm, corresponding to a tip speed of 3.42 m/s, with a rotor diameter of 172 mm and a feed rate of 4 kg/h. Additionally, the gap between the cutting and stator knives was adjusted to a size of less than 200 µm to gain optimal milling result.

2.2.2 Wet Milling

A Fryma MZ 80 wet rotor mill (FrymaKoruma, Rheinfelden, Germany) with a motor power of 4 kW was employed for further particle size reduction. The mill operated at a rotational speed of 2900 min^{-1}, corresponding to a tip speed of approximately 12 m/s. Comminution was performed in a circular mode utilizing a coarse-toothed rotor. A stirrer was integrated into the mill's feed tank, with the speed set to 150 rpm and regulated via the IKA Eurostar 60 control system (IKA, Staufen im Breisgau, Germany). The gap width was adjusted to 350 µm, and a solids load of 5 wt% was tested. Prior to milling, the textile suspension, along with all ingredients except the enzymes, was soaked for 30 minutes and subsequently heated to 50 °C. All ingredients used in the experimental setup are described in detail in chapter 2.3.2). Both the feed tank and the feed pipe were connected to a MAGIO MS-BC 4 circulation thermostat (Julabo, Seelbach (Schütter), Baden-Württemberg, Germany) to maintain temperature control of the suspension.

To evaluate the effects of mechanical treatment with and without enzymes, various processing options were assessed using both cotton textile waste and white cotton. The experiment started with the addition of enzymes, while prior milling was consistently conducted without the use of enzymes. The RAging cycles employed included 1 minutes of milling followed by 29 minutes of resting, as well as a cycle of 0.16 min of milling followed by 29.84 minutes of resting. The throughput of the suspension ranged from 3.29 t/h to 6.43 t/h, with variations arising from differing process parameters. The passage number n_p (-) was defined as milling time t_m , divided by the time required a fiber to pass through feed tank and the mill once t_p. It corresponds to the average residence time of the wet rotor mill for pumping the suspension once and is calculated by dividing the volume V (m^3) of the suspension by the throughput $\dot{V}\ (\frac{m^3}{s})$:

$$n_p = \frac{t_m}{t_p} = \frac{t_m \cdot \dot{V}}{V}. \tag{1}$$

The number of passages per milling step varied significantly, ranging from 110 during milling (10 minutes) to 11 during RAging (1 minute), and 1.8 for the shorter duration (0.16 minutes), depending on the specific combination of milling and enzymatic degradation. Detailed calculations can be found in previous publications [1]. Power consumption of the wet rotor mill was monitored using the MCR-S-10-50-UI-DCI current transducer (Phoenix Contact, Blomberg, Germany), with measurement values recorded at ten-second intervals. The power intake (*P*) of the mill was calculated by multiplying the measured electrical current (*I*) by the voltage (*U*) and the factor of 0.85 ($\cos(\varphi)$). The specific energy (*e*) was determined by multiplying the power intake (*P*) by the time (*t*) and dividing by the mass of the suspension (*m*):

$$P = \sqrt{3} \cdot U \cdot I \cdot \cos(\varphi). \tag{2}$$

The specific energy *e* was determined using the power intake *P* multiplied by the time *t* and divided by the mass of the suspension *m*:

$$e = \frac{P \cdot t}{m}. \tag{3}$$

2.3 Analysis of Product Properties

2.3.1 Determination of Enzyme Activity

The enzyme assay developed by Adney and Baker [2] was utilized to assess cellulase activity. A total of 50 mg of filter paper (Whatman, Cytiva, Marlborough, Massachusetts, USA) was subjected to digestion with various concentrations of cellulase. The enzyme preparation was diluted in 2 mL of a 50 mmol/L citrate buffer and incubated at 50 °C for a duration of 60 minutes. The resulting glucose molecules were quantified using a UV-VIS spectrometer (LAMBDA Bio+, PerkinElmer, Shelton, Connecticut, USA), with absorbance measured at 540 nm. The glucose concentration obtained was employed to establish a standard curve correlating enzyme concentration with the quantity of released glucose equivalents. Ultimately, the filter paper units (FPU) were calculated, representing the enzyme amount required to generate 2 mg of reducing sugars. Due to the utilization of multiple batches of cellulase blends, the activity of each batch was found to range between 260 FPU/mL and 290 FPU/mL.

2.3.2 Enzymatic Hydrolysis

A commercial cellulase formulation, Cellic CTec2 (Novonesis, provided by Sigma-Aldrich, SAE 0020-50mL) was employed for the enzymatic hydrolysis of cotton textiles. The hydrolysis process was conducted in a 50 mM citrate buffer at pH 4.8 and a temperature of 50 °C in a feed tank connected to a wet rotor mill (FrymaKoruma, Rheinfelden, Germany). An enzyme concentration of 25 FPU per gram of substrate were utilized. The textile loadings in suspension (wt%) were used at 5 % cotton textile. To each trial, 4750 mL of 50 mmol/L citrate buffer and 1 g/L of polyethylene glycol (PEG 6000, AnalytiChem, Eschborn, Germany) were added. Polyethylene glycol (PEG) was included in all experimental trials in this study, as it is a cost-efficient additive known to improve the efficiency of enzymatic hydrolysis, as supported by previous research [3,4]. The desired enzyme concentration was introduced at the beginning of the experiment. Duplicate samples of 400 µL were collected every thirty minutes over a period of 6 hours.

To assess potential enzymatic inhibition and influence of milling on textile fibers, hydrolysis experiments were conducted using an incubator (INFORS Multitron, Bottmingen, Switzerland) set to 150 rpm with a 25 mm shaking diameter. The enzymatic reactions were carried out with a textile substrate loading of 2.5 % (w/w) and 5 % (w/w), and an enzyme concentration of 25 and 50 FPU per gram of substrate. Prior to hydrolysis, the textile material was milled at a solids concentration of 5 % using a gap width of 350 µm and a specific energy input of 85 kWh/t. The milled suspension was centrifuged at 5000 g for 10 minutes (Eppendorf 5430, Hamburg, Germany), and the resulting solids were dried at 50 °C for three days in a drying oven (Heraeus, Hanau, Germany). Dried samples were then weighed into 50 mL falcons. Each sample was supplemented with 39 mL of 50 mmol/L citrate buffer and 1 g/L polyethylene glycol (PEG 6000, AnalytiChem, Eschborn, Germany). Enzymes were added at the beginning of the experiment. Aliquots of 400 µL were collected at 0, 3, 6, and 24 hours for subsequent analysis.

All samples were heated to 99 °C for 5 minutes in an Eppendorf (1000 rpm, deflection: 3 mm) and then centrifuged at 11603 g (Hettich Micro 22R centrifuge, Tuttlingen, Germany). The resulting samples were filtered and transferred to Greiner microtiter plates (Greiner Bio-One GmbH, Frickenhausen, Germany). The plates were then cooled to 4 °C until the glucose concentration was measured using a photometric assay with a CEDEX device (Roche, Basel, Switzerland). It is noteworthy that Cellic CTec2, as a commercial enzyme solution, contains a considerable amount of glucose. This glucose content was quantified using reference samples that did not contain textile fibers and was subsequently subtracted from the total measurements.

2.3.3 Glucose Measurement

For the determination of glucose concentration, a Cedex Bio Analyzer 3D Lab (Roche, Basel, Switzerland) equipped with software version 3.0 was utilized. Additional details can be found in earlier publications [1]. Within the scope of this study, the glucose yield ($Y_{Glucose}$), expressed as a percentage, is defined as the quantity of glucose released ($ß_{Glucose}$, in g/L) divided by the initial cellulose concentration ($ß_{Cellulose}$, in g/L), multiplied by a factor of 1.111, which represents the theoretical glucose concentration [5–7]. The factor 1.111 arises from the conversion of an anhydroglucose unit in cellulose (162.14 g/mol) to free glucose (180.16 g/mol) during hydrolysis, accounting for the addition of a water molecule:

$$Y_{\text{Glucose}} = \frac{ß_{\text{Glucose}} \cdot 100\ \%}{ß_{\text{Cellulose}} \cdot 1.111}. \tag{4}$$

Figure S 1 Effect of mechanoenzymatic processing on morphology. Five processing conditions were applied to cotton textile waste: (A) No Milling (NoMi), (B) RAging cycle 1 (RA), (C) Milling + RAging cycle 1 (MiRA 1), (D) Milling + RAging cycle 2 (MiRA 2), and (E) Crude Textile. SEM images after a 6-hour trial period. Scale bar: 10 µm.

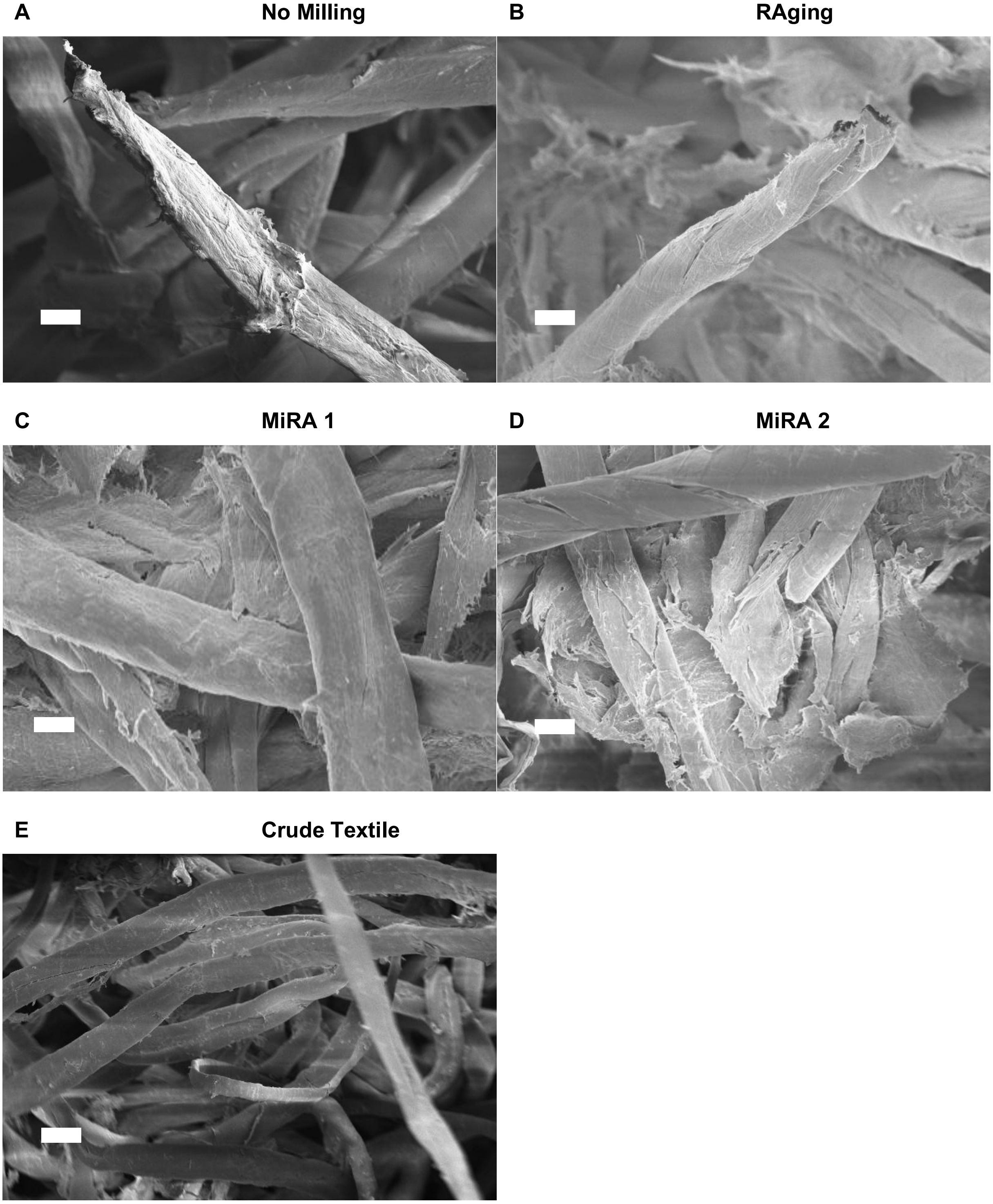

References

[1] M. M. Schaake, O. Pikhard, M. Bross, T. May, Z. C. Hua, L. Schmidt, F. K. Jaeger, A. Liese, S. Heinrich, “Optimizing mechanical pretreatment of cotton textile waste to enhance enzymatic hydrolysis” *Waste Management* **2025**, *204*, 114967.

[2] B. Adney, J. Baker, “Measurement of Cellulase Activities: Laboratory Analytical Procedure (LAP); Issue Date: 08/12/1996” *Technical Report* **2008**.

[3] J. Li, S. Li, C. Fan, Z. Yan, “The mechanism of poly(ethylene glycol) 4000 effect on enzymatic hydrolysis of lignocellulose” *Colloids and Surfaces B: Biointerfaces* **2012**, *89*, 203–210.

[4] V. Pihlajaniemi, A. Kallioinen, M. H. Sipponen, A. Nyyssölä, “Modeling and optimization of polyethylene glycol (PEG) addition for cost-efficient enzymatic hydrolysis of lignocellulose” *Biochemical Engineering Journal* **2021**, *167*, 107894.

[5] A. Goshadrou, K. Karimi, M. Lefsrud, “Characterization of ionic liquid pretreated aspen wood using semi-quantitative methods for ethanol production” *Carbohydrate Polymers* **2013**, *96*, 440–449.

[6] A. Jeihanipour, K. Karimi, C. Niklasson, M. J. Taherzadeh, “A novel process for ethanol or biogas production from cellulose in blended-fibers waste textiles” *Waste Management* **2010**, *30*, 2504–2509.

[7] S. Nikolić, V. Lazić, Đ. Veljović, L. Mojović, “Production of bioethanol from pre-treated cotton fabrics and waste cotton materials” *Carbohydrate Polymers* **2017**, *164*, 136–144.
